# Recent Advances in Green Synthesis of Ag NPs for Extenuating Antimicrobial Resistance

**DOI:** 10.3390/nano12071115

**Published:** 2022-03-28

**Authors:** Simerjeet Parmar, Harwinder Kaur, Jagpreet Singh, Avtar Singh Matharu, Seeram Ramakrishna, Mikhael Bechelany

**Affiliations:** 1 Department of Biotechnology, Sri Guru Granth Sahib World University, Fatehgarh Sahib 140406, India; simerparmar36@gmail.com (S.P.); harwinderkaur2412@gmail.com (H.K.); 2 Department of Chemical Engineering, Chandigarh University, Gharuan, Mohali 140413, India; 3 University Centre for Research and Development, Chandigarh University, Gharuan, Mohali 140413, India; 4 Department of Chemistry, Green Chemistry Centre of Excellence, University of York, York YO10 5DD, UK; avtar.matharu@york.ac.uk; 5 Department of Mechanical Engineering, Centre for Nanotechnology & Sustainability, National University of Singapore, Singapore 117575, Singapore; seeram@nus.edu.sg; 6 Institut Européen des Membranes, IEM, UMR 5635, University of Montpellier, ENSCM, CNRS, 34000 Montpellier, France

**Keywords:** antimicrobial resistance, silver NP’s, green synthesis, environmental

## Abstract

Combating antimicrobial resistance (AMR) is an on-going global grand challenge, as recognized by several UN Sustainable Development Goals. Silver nanoparticles (Ag NPs) are well-known for their efficacy against antimicrobial resistance, and a plethora of green synthesis methodologies now exist in the literature. Herein, this review evaluates recent advances in biological approaches for Ag NPs, and their antimicrobial potential of Ag NPs with mechanisms of action are explored deeply. Moreover, short and long-term potential toxic effects of Ag NPs on animals, the environment, and human health are briefly discussed. Finally, we also provide a summary of the current state of the research and future challenges on a biologically mediated Ag-nanostructures-based effective platform for alleviating AMR.

## 1. Introduction

Microbial infections cause a variety of chronic diseases and account for around 10 million deaths each year, most of which occur in tropical nations. The developed world is not immune either. Antibiotics have been utilized to treat bacterial infections because of their cost-effectiveness and potent results. Antibiotic abuse and overuse, on the other hand, have aided the development and spread of resistance mechanisms among bacteria, resulting in the creation of multidrug-resistant (MDR) microorganisms. The annual cost of multidrug-resistant (MDR) microorganisms in the United States is estimated to be around $20 billion [1,2]. Clinicians have no effective alternative to treat infected patients due to the development of multidrug-resistant bacteria and super bugs. Antibiotic overuse promotes the emergence and evolution of strains with a wide range of genotypic and phenotypic characteristics, jeopardizing antibiotics’ therapeutic value. The ability to form biofilms is one of the most efficacious mechanisms available, which is associated with about 65–80% of human infections. Bacteria can form a biofilm on a surface and proliferate as a colony where cells aggregate together and surround themselves with a self-produced extracellular matrix that makes cells 100–1000 times less susceptible to antibiotics than planktonic cells [3]. Antimicrobial drug resistance has been identified by the World Health Organization as one of the top three global public health problems. According to WHO data, nearly 80% of MDR or XDR bacteria are caused by antibiotic abuse and overuse, and these infections are linked to serious side effects [4]. As a result, alternative therapeutic approaches for microbial pathogens are required. Nanoparticles have vital biological uses, such as antibacterial, drug delivery, and bioimaging, because of their unique physicochemical characteristics, such as high specific surface area, optical properties, antimicrobial activity, catalytic activity, electronic properties, and magnetic properties [5,6,7,8,9]. Silver exhibits strong toxicity against a wide range of microbes (anti-bacterial applications). Silver compounds are known to be effective against both aerobic and anaerobic bacteria. Silver precipitates bacterial cellular proteins and blocks the microbial respiratory chain system. Before silver nanoparticles, silver nitrate was used as an effective antibacterial agent that follows various mechanisms of action, including direct penetration inside the bacteria, inhibition of DNA by interaction with bacterial membrane proteins, and an attack on an electron transport chain in mitochondria [5,10,11].

Although there have been numerous papers written on this topic in the past [5,10,12] and recently [10], this article presents the most up-to-date information with year-by-year comparisons that are no more than three years old. Furthermore, it presents a toxicity evaluation of Ag NPs with crucial future difficulties. As a result, given its comprehensive scope and up-to-date analysis, this article will be extremely useful and serve as an effective single platform for readers and researchers interested in the subject of nanotechnology.

### Methods of AgNPs Synthesis

**Figure 1:**
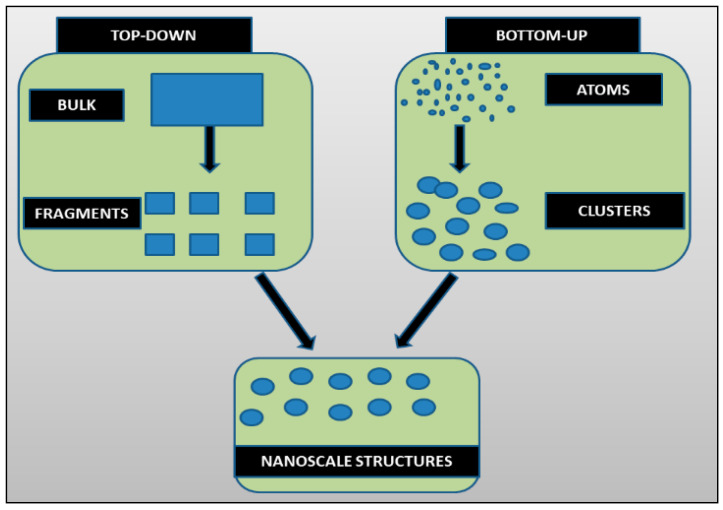
“Top-down” and “bottom-up” approaches for the synthesis of nanoparticles.

Traditionally, two approaches are used to synthesize nanomaterials: i. ‘Top-down’ and ii. ‘Bottom-up’ (Figure 1). The top-down approach generates nanoparticles using size reduction of bulk materials techniques, i.e., pulse laser ablation, pulse wire discharge method evaporation—condensation, ball milling, etc. In the bottom-up approach, chemical and biological methods are used to synthesize NPs by a self-assembly phenomenon of atoms to new nuclei that grow in the particles of nanoscale dimensions [11].

In physical processes, the synthesis of AgNPs is achieved by evaporation–condensation method, which operates tube furnace at atmospheric pressure. AgNPs are also being produced with laser ablation of metallic bulk materials in solution. The biggest advantage of laser ablation over the conventional method is the removal of chemical reagents in solutions. Hence, pure colloids can be created by this technique, which will be valuable for additional applications [5]. Furthermore, for the preparation of Ag NPs, physical vapor deposition methods such as sputtering are considered to be a green and safe option [12,13].

**Figure 2:**
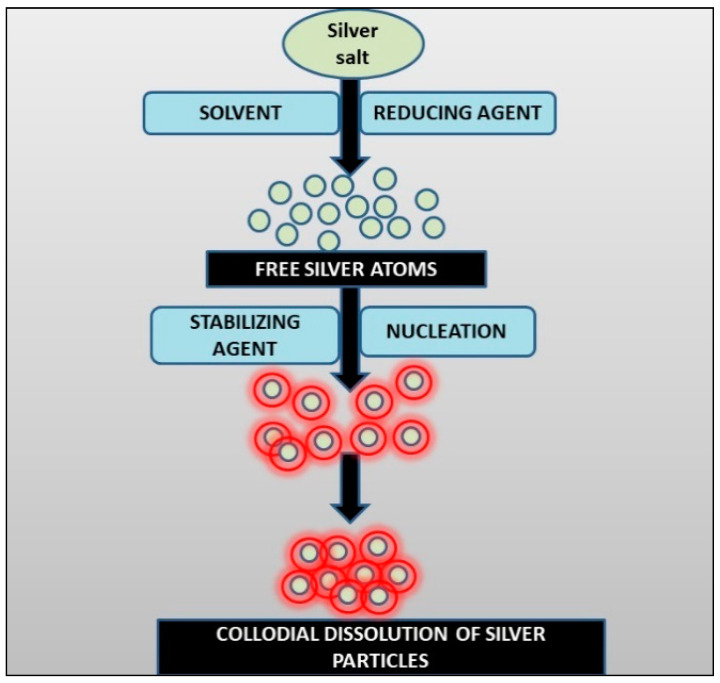
Schematic of Ag-NPs synthesis by the chemical reduction method.

Chemical reduction of metal salt solutions is one of the most common methods for the synthesis of AgNPs [5]. For the most part, the chemical synthesis for AgNPs has three fundamental components: i. reducing agents; ii. precursors; and iii. stabilizing/capping agents. The resultant nanoparticles tend to nucleate and grow to produce a colloidal solution (Figure 2) [14].

The use of high temperatures, hazardous chemicals, and pressure, and the formation of dangerous by-products, are all downsides of chemical-physical approaches for AgNPs synthesis, necessitating the search for safer alternatives. The biological production of NPs is gaining importance in this way. This list of disadvantages is continuous with difficult separation procedures, high pressure, and energy requirement. Their large-scale production is difficult, chemical purification of nanoparticles is needed, and controlling the accumulating parameters is also hard to achieve [15,16].

To address these issues, scientists have been looking for greener alternatives, such as naturally occurring sources and their products, that can be utilized to synthesize NPs. The development of biological methods for the synthesis of Ag-NPs is becoming a major branch of nanotechnology. The bioreduction of the Ag^+^ ions, which utilizes nitrate, can be attained by metabolic processes. Nitrate is consumed as a main source of nitrogen by cyanobacteria, as described in the following equations:

NO_3_^−^ + 2H^+^ + 2e^−^ = NO_2_^−^ + H_2_O

NO_2_^−^ + 8H^+^ + 6e^−^ = NH_4_^+^ + 2H_2_O

It is suggested that Ag^+^ ions could also be reduced by an intracellular electron donor [5].

**Figure 3:**
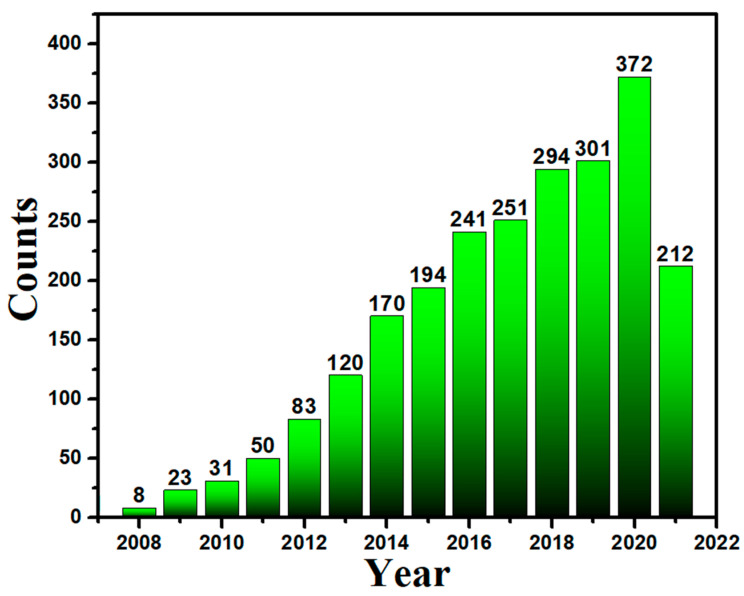
A histogram shows the frequency of paper publication on biological approaches for Ag NPs.

The biosynthesis approach is cost-efficient and environmentally friendly. The purpose of this review article is to disseminate information about the significance of biosynthesized AgNPs to mitigate the contemporary AMR issue in the pharmaceutical industry. Moreover, this study also involves the detailed concepts of biological approaches and explores the advantages of green approaches over the physical or chemical approaches, which foster one to follow these cost-effective and benign biological approaches. We also collected paper publication data from PubMed (Figure 3), which indicates the continuous increase of interest of researchers in biological approaches for Ag NPs.

## 2. Synthesis of Ag-NPs via Biological Methods

Green synthesis can be divided into two categories:

(a) The use of microorganisms such as yeasts, bacteria, fungus, and actinomycetes in the production of chemicals; and
(b) Extract of different parts of plants.

The next sections explain biological synthesis employing bacteria, fungus, and plant extracts.

### 2.1. AgNPs Synthesis via Bacteria

Bacteria can reduce metal ions and can produce NPs. Therefore, there are numerous bacterial species that are being exploited for this purpose [17], for example:

- *Geobacter spp*., *Arthrobacter gangotriensis*;
- *Bacillus cereus*, *Antarctica Bacillus amyloliquefaciens*;
- *Corynebacterium sp. SH09*, and *Shewanellaoneidensis*;
- *Pseudomonas proteolytica*, *Aeromonas sp. SH10 Phaeocystis*;
- *Escherichia coli*, *Lactobacillus case*;
- *Bacillus cecembensis*, *Enterobacter cloacae*, and *Bacillus indicus*.

There are two methods for Ag NPs synthesis using microbes: i. intracellular method and ii. extracellular method. The synthesis of Ag NPs via different bacterial species is summarized in Table 1.

#### 2.1.1. Intracellular Method and Mechanism

The intracellular approach involves the deposition of silver within the cell, which initiates the synthesis of Ag NPs while maintaining microbial growth. The live cells with nanoparticles are retrieved after the bacteria have grown to their maximum potential. To secrete the NPs, the collected cell requires special treatment. Bacterial extracellular secretions are isolated and employed in the synthesis mechanism during the extracellular process. Microorganisms that are resistant to silver ions are more likely to make AgNPs, and the mechanism of resistance differs depending on the organism [46].

**Figure 4:**
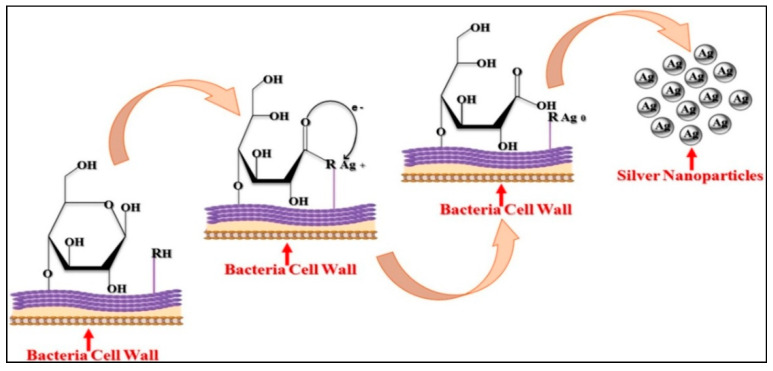
Non-enzymatic intracellular synthesis of AgNPs by bacteria [47].

To exemplify intracellular synthesis, LactobacillusA09, where Ag^+^ reduction took place on the bacterial cell surface, was used to explain intracellular synthesis. Lactobacillus A09 bacteria have many anionic surface groups on their cell walls, which act as Ag-NPs ions biosorption sites. The pH of the solution gradually fell after Ag^+^ adsorption on Lactobacillus A09, and competition between proton and silver, which binds to negatively charged sites, occurred. Due to an increase in pH, the monosaccharide rings in bacterial cell walls are disrupted, and the oxidized monosaccharide rings are converted to open-chain aldehydes. Negatively charged Ag^+^ adsorption sites on the cell surface originate from the dissociation of protons from protonated anionic functional groups (–RH). While forming the aldehyde group, the two electrons are released from alcohol, which can reduce Ag^+^ ions to elemental Ag^0^. Few of the steps in the bacterial production of Ag-NPs are mediated by the opening of the glucose ring (Figure 4) [47].

#### 2.1.2. Extracellular Method and Mechanism

**Figure 5:**
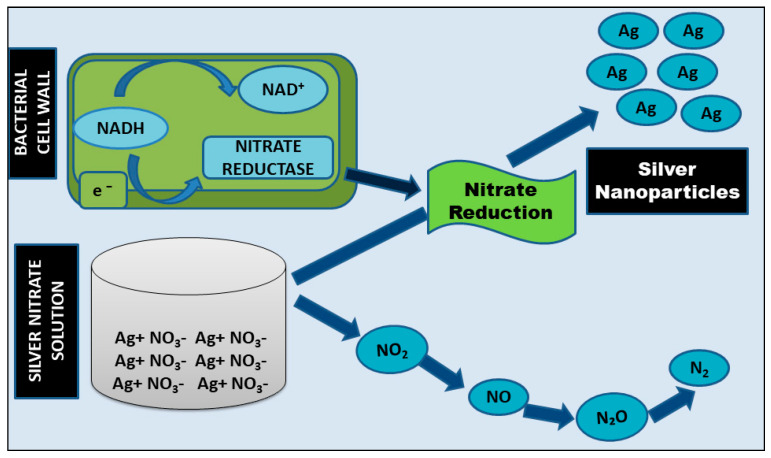
Extracellular enzymatic synthesis of Ag-NPs by bacteria.

Streptomyces sp.LK3 and Bacillus licheniformis are two bacteria that generate Ag-NPs by reducing silver ion (Ag^+^) via an extracellular process driven by reduced nicotinamide adenine dinucleotide (NADH)-dependent nitrate reductase. Nitrate ions (NO_3_) in silver nitrate (AgNO_3_) are reduced to nitrite (NO_2_) by taking two protons and then releasing two electrons and water during the reduction process. Elemental silver is formed when the electrons liberated in this process are transferred to the silver (Ag^0^). This method may be reliant on electron transport channels and enzymatic metal reduction activities (Figure 5) [48].

### 2.2. Synthesis by Using Fungi

The employment of fungi in the production of metal/metal oxide NPs is also a reasonably methodical strategy for producing monodispersed NPs with distinct morphologies (Table 2). For the generation of metal and metal oxide nanoparticles, fungi are good biological agents, due to the presence of numerous intracellular enzymes [9]. The advantages of fungi over other microbes include large production of proteins and enzymes, and fast and sustainable synthesis of nanoparticles. Fungi are frequently utilized as a stabilizing and reducing agent due to their heavy metal tolerance and propensity to absorb metals. Moreover, the production of fungi on a large scale is easy (“nano factories”) and can produce nanoparticles of desired size and morphology [40].

#### Mechanism of Synthesis

The specific mechanism of the synthesis of AgNPs using is still not known. However, in general, the mechanism can be extracellular or intracellular. In the mycelial culture, the metal precursor is incorporated in the biomass, in the intracellular approach. Following synthesis, Ag-NPs extraction is required. To disrupt the biomass and liberate the nanoparticles, the extraction technique includes centrifugation, chemical treatment, and filtering.

**Figure 6:**
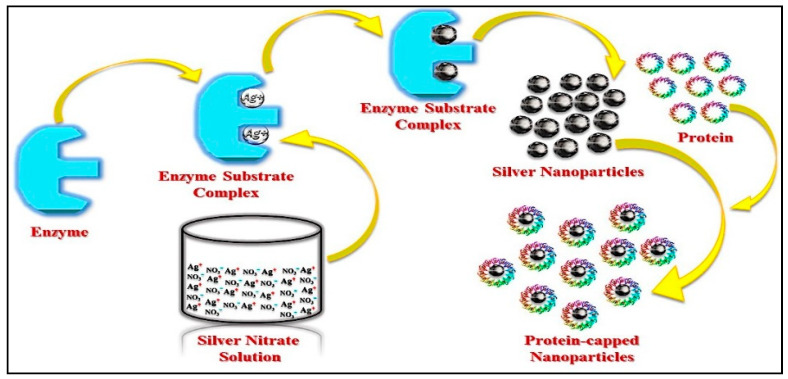
Fungal synthesis of Ag-NPs [47].

In extracellular synthesis, metal precursor is added to a filtrate that solely includes fungal biomolecules in extracellular synthesis, leading to the generation of free NPs in the dispersion solution. This method is widely utilized, and it eliminates the requirement for AgNPs to be extracted from cells [40]. Extracellular nanoparticle formation is found to follow processes in which Ag ions are converted to elemental silver (Ag^0^) by an enzyme present in the fungal filtrate. [40]. Mainly reductases are involved in the fungal NP synthesis. For example, Aspergillus flavus releases a 32-kDa reductase protein that lowers Ag^+^ ions during the formation of Ag-NPs, as described in Figure 6 [47].

In comparison to bacteria, several studies have demonstrated the appropriateness and possibility of employing fungus for large-scale NP synthesis. AgNPs were recently created utilizing A. flavus fungus and antibiotics to increase biocidal efficacy against multidrug-resistant bacteria, resulting in antibiotics coupled with AgNPs being more effective [11].

### 2.3. Synthesis of AgNPs by Using Plants

The utilization of plant extracts for AgNPs synthesis provides a number of merits over chemical, physical, and microbiological approaches. The removal of hazardous reducing and capping chemicals, radiation and high temperature, microbial strain, and expensive media for microbial growth are just a few of the positives. The time required for the synthesis of AgNPs is also less using plants. For example, when compared to microbes, neem leaf extract is faster at removing metals. Plant-synthesized AgNPs remain constant for a longer time and have application in the biomedical field. Different factors such as temperature, pH, reaction period, changing ratio, and concentration of plant extract and precursor (AgNO_3_) can be controlled to synthesize AgNPs on a large scale with various shapes and sizes, which is difficult or impossible to do in microbial synthesis (Table 3) [78].

#### Mechanism of Synthesis

Plants have various biomolecules (lcarbohydrates, proteins, terpenoids, flavonoids, flavones, terpenes, phenolics, polysaccharides, saponins, tannins, alkaloids, and coenzymes) have the greatest capacity to convert metal salt to NPs. Several plants are used for synthesis, including Coriander (*Coriandrum sativum*), Oat (*Avena sativa*), alfalfa (*Medicago sativa*), aloe vera (*Aloe barbadensis Miller*), Neem (*Azadirachta indica*), Mustard (*Brassica juncea*), and lemongrass (*Cymbopogon fexuosus*), Tulsi (*Ocimum sanctum*), and Lemon (*Citrus limon*).

**Figure 7:**
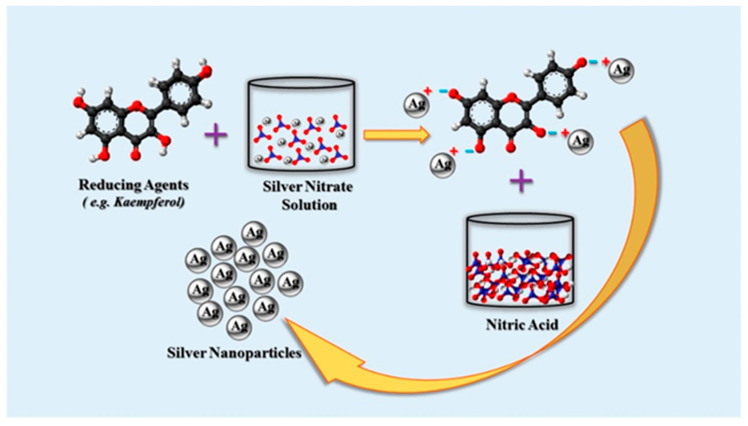
Plant-based synthesis of Ag-NPs by reaction of AgNO_3_ with phytochemicals [47].

Plant extracts have the ability to serve as both reducing and stabilizing agents during the production of AgNPs. Phytochemicals include functional groups that can reduce Ag^+^ ions to AgNPs, such as hydroxyl, amino, ketone, carboxyl, and aldehyde groups. For instance, the flavonoids naringin and kaempferol, as well as their glycosides, are found in Punica granatum peel extract. All substances with hydroxyl (–OH) groups can cause Ag^+^ ions to be reduced, resulting in the creation of AgNPs, as illustrated in Figure 7 [47,48].

### 2.4. Key Factors for Efficient, Economical, and Reliable Preparation of AgNPs 

The proportions of plant extracts and metal salts, ambient duration, temperature, pH, and other parameters all play a role in the quick, sustainable, and scalable manufacturing of AgNPs. These variables also influence the form and size of obtained NPs.

Temperature and metal salt content impacted extracellular production of AgNPs employing culture supernatant of *Pseudoduganella eburnea MAHUQ-39*, as per Huq [127]. The optimal conditions for the efficient and sustainable formation of AgNPs utilizing *P. eburnea* were determined to be 30 °C temperature, 1 mM AgNO_3_ (final concentration), and 24 h incubation duration. The impact of temperature, ionic strength, and contact time on the ecofriendly synthesis of AgNPs via bark extracts of *A. indica* and *F. benghalensis* was studied by Nayak et al. [128], who found that an 80 °C temperature, a pH of 10, and a 30 min incubation time are the best conditions for quick and reliable production.

Mittal et al. [129] extracted the natural herb *Potentilla fulgens* for the ecofriendly synthesis of AgNPs and discovered that assorted physicochemical variables such as plant extract and metal ion concentration levels, incubation duration and temperature, and the pH of the reaction time all had a significant impact on the rate of synthesis as well as the shape, size, and yield. They tested various plant extract concentrations (1 to 200 mg in 50 mL water) and discovered that 4 mg extract in 50 mL water produced the maximum quantity of AgNPs. They additionally employed different concentrations of AgNO_3_ ranging from 0.5 to 5 mM and discovered that the production of AgNPs enhanced as the AgNO_3_ amount grew from 0.5 to 1 mM, after which the absorbance dropped again. They discovered that 45 °C is the optimal temperature for the highest yield, and that higher temperatures improved the rate of synthesis of finer NPs. The pH of the precursor solution had an impact on the synthesis. They discovered that finer NPs developed at an alkaline pH, while bigger NPs emerged at an acidic pH.

Hamouda et al. [130] used an aqueous extract of *Oscillatoria limnetica* to analyze the influence of plant extracts and AgNO_3_ doses on the synthesis of NPs. They found that the quantities of the aqueous leaf extract of Oscillatoria limnetica and AgNO_3_ affected the size and shape of obtained AgNPs. These characteristics have a substantial impact on microbe-mediated synthesis, just as they do on plant-mediated synthesis.

Much additional recent research has shown the influence of plant extract and metal salt concentration, incubation duration, temperature, and pH on the quick and stable synthesis of homogeneous AgNPs with a high yield employing both plants and microorganisms [131,132,133].

## 3. Antimicrobial Activity of Ag NPs

The fast growth of drug resistance to new antibiotics, as well as rapid mutations, has resulted in the creation of antimicrobial substances and alternate therapies [46]. It is reported that more than 70% of infections by bacteria are resistant to one or more antibiotics that are used to treat the infection. Metals such as copper (Cu), titanium (Ti), silver (Ag), gold (Au), and zinc (Zn) are known to show antimicrobial activity [134]. AgNPs can cause structural damage, produce ROS, disrupt DNA replication, and react with the thiol group of enzymes, among other biocidal effects (Table 4). The antagonistic AgNPs influence the enzymes and proteins of bacteria regardless of their Gram characteristics, according to these investigations. Antibiotics that target a specific method of microbial suppression, on the other hand, do not have this problem [135]. Silver compounds are known to be effective against aerobic and anaerobic bacteria by precipitation of cellular proteins of bacteria, and the microbial respiratory chain system has been blocked. Antiviral activity of AgNPs with a large surface-to-volume ratio (size ≤ 100 nm) against HIV-infected cells has been demonstrated [46]. They are also effective against multidrug-resistant organisms such as Streptococcus pyogenes, methicillin-resistant Staphylococcus aureus (MRSA), *Escherichia coli*, *Pseudomonas aeruginosa*, and vancomycin-resistant *Staphylococcus aureus* [46].

### 3.1. Mechanism of Action

Antibacterial research on silver nanoparticles is generally done in vitro on solid media or liquid cultures of microorganisms. The antibacterial mode of action of AgNPs, as well as their influence on other bodily components, must be investigated in vivo. The antibacterial activity of AgNPs produced from plant extracts has been well demonstrated; however, the specific mechanism is unknown [97].

**Figure 8:**
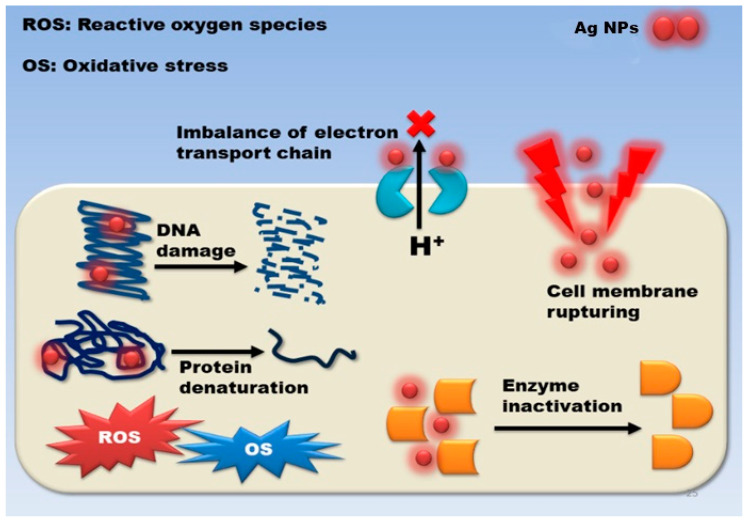
Silver nanoparticles’ antibacterial mechanism of action is described in general terms [79].

Despite this, a lot of studies have attempted to figure out how they work, and three distinct processes have been postulated so far: damage to cell membranes and cell walls penetrating and damaging cells within cells oxidative stress, as depicted in Figure 8.

### 3.2. Damage to the Cell Wall and Membrane

The fundamental purpose of the cell wall and membrane is to protect microorganisms from external threats and to maintain homeostasis while allowing nutrients to be transported within the cell. AgNPs exhibit high antibacterial activity against Gram-negative bacteria as compared to Gram-positive bacteria because the peptidoglycan layer present in the Gram-positive bacterial cell wall, which acts as a natural barrier, is thick and thus averts the diffusion of the NPs. Due to adhesion between AgNPs and microorganisms, interaction occurs between the microbial cell wall and the surface of the Ag-NPs. The electrostatic attraction between the negative charge on the microbial cell membrane and the positive or less negative charge on AgNPs determines the outcome of this interaction. Following such attraction and contact, the NPs produce morphological changes in the membrane’s structure, resulting in membrane permeability and respiratory functions being disrupted by membrane depolarization, which ultimately disrupts cell integrity and cell death. It was found that when membrane permeability increases and the cell wall is disrupted, cellular content such as DNA, enzymes, organelles, ions, metabolites, and the energy reserve seeps into the environment. As a result, damage to cell membranes and cell walls, intracellular penetration and damage, and oxidative stress, as depicted in Figure 8 [46].

### 3.3. Intracellular Penetration and Damage

AgNPs can permeate the cell and affect essential activities such as DNA and protein interaction, depending on the degree of membrane damage. One of the recognized mechanisms for AgNPs antibacterial action is silver ion release from the NPs, which has a detrimental impact on microorganisms. Silver ions were shown to cause the transition of bacteria’s DNA from a naturally relaxed state to a compacted one, in which the molecule of DNA loses its reproduction ability. Furthermore, X-ray examination reveals the presence of sulphur, indicating that silver ions interact with protein thiol groups, resulting in the inhibition of enzyme function. AgNPs can cause DNA degradation and/or denaturation, in addition to changing their configuration. Ag^+^ ions are also attached to DNA via physical attractions and interact with the nucleoside component of the nucleotide, according to research. The base-pairing inside complimentary strands is altered as a result of the hydrogen bond breakage [46].

The intracellular action of Ag NPs is not limited to DNA destruction. The effects of Ag NPs on proteins and protein synthesis have been discovered through proteomic investigations. Previous research has shown that silver nanoparticles and Ag^+^ ions formed from AgNPs are reactive with protein thiol groups. Cysteine amino acids are found with thiol or thiolate groups as their functional group. Cysteine is an inadequate amino acid; however, it is a highly conserved residue in functional protein locations. It is important in biological processes because of its high-affinity metal-binding capability, nucleophilic participation in catalytic events, and ability to form disulfide bonds, which is essential for protein folding and 3-D structure.

### 3.4. Oxidative Stress

The term “reactive oxygen species” (ROS) refers to oxygen-containing compounds with high redox potential. When circumstances are normal, the generation of ROS inside the cell is balanced, as is its antioxidant capacity. However, due to an imbalance between the antioxidant mechanism and the inappropriate release of ROS, the redox balance of cells favors oxidation, resulting in oxidative stress. AgNPs cause cellular oxidative stress, and cells respond by exhibiting defensive responses that include enzymatic and non-enzymatic defense mechanisms to counteract this stress. When oxidative stress overwhelms these defensive systems, ROS and free radicals cause damage to the cell wall and macromolecules, including proteins, lipids, and DNA.

DNA damage includes deletions, mutations, single and double-strand breaks, adduct accumulation, and protein cross-linking. Studies have shown that oxidation-mediated DNA fragmentation occurs following exposure to metal oxide NPs. Cells strive to repair damaged DNA in response to DNA damage. Failure to repair can lead to cell death. There is also a possibility of the generation of ROS, which is mediated by Ag^+^ ions produced by AgNPs, which may disrupt the bacterial electron transport chain as well as proton motive force. This leads to enzyme inhibition involved in the reactions. Researchers also found that, in addition to the breakdown of membrane functions, ROS generation also causes protein leakage through increased membrane permeability. These leaked proteins by the cells interact with AgNPs, which eventually leads to cell death. Oxidative stress can change gene expression, in addition to its direct effects on cell walls and components. On the treatment of Pseudomonas cells with AgNPs, translation of ribosomal proteins S2 and L9, alkyl hydroperoxide reductase C (AhpC), keto-hydroxyglutarate aldolase (KHGA), and thiol-specific antioxidant (TSA) was deemed to be overexpressed.

Thus, the essential aspect of NPs is their method of action, which is influenced by their size; dissolving efficiency; ionic strength of the medium; synthesis and treatment variables; and the kind of stabilizing agent used.

AgNPs have been shown to impede protein expression along with cell wall production in the literature, providing strong evidence for protein breakdown of the exterior cell surface and increased ATP permeability, leading to apoptosis [153].

Furthermore, the size and morphology of AgNPs were shown to boost the production of Ag^+^ ions due to their larger surface area, thus influencing their potency towards microbial illness. AgNPs’ aqueous solubility significantly affects their antibacterial activity. The potential efficacy might be boosted if the aqueous solubility is significant [154]. AgNPs smaller than 10 nm are thought to be capable of directly penetrating cell walls, entering bacterial cells, and causing cell lysis [155]. As a result, the findings might be useful in determining if AgNPs can be used as an alternative antibacterial agent to prevent dangerous microorganisms and alleviate microbial disease illnesses.

## 4. Toxicology of Silver Nanoparticles to Human Health

Although nanotechnology has been exploited in a large number of commercial products overall the world recently, there is still a lot of information lacking regarding the increase of human, animal, and ecological exposure to AgNPs and their short and long-term potential lethal effects [156]. Silver can enter the human body and shows lethal effects on human health. Previous literature indicated that Ag^+^ ions alter the cell membrane permeability to K^+^ and then to Na^+^. This reduces the mitochondrial function or ATP activity and increases membrane leakage [156,157]. Silver nanoparticles were found to have a significant cytotoxic effect on peripheral blood mononuclear cells (PBMCs) when levels were over 15 ppm, and Phyto-haemagglutinin-induced cytokine production was remarkably inhibited by silver nanoparticles [157]. Additionally, lactate dehydrogenase (LDH) leakage was notably elevated in cells exposed to AgNPs (10–50 µg mL^−1^). A study proved the significant decrease in reduced glutathione (GSH) level, and increase in reactive oxygen species (ROS) levels, which signifies that AgNPs cytotoxicity (15, 100 nm) is mediated by oxidative stress in liver cells. Silver nanoparticles also exhibit severe effects on the male reproductive system. Studies suggest that silver nanoparticles can cross the blood-testes barrier and be deposited in there. They show potential adverse effects on sperm cells. Silver also accumulates and shows some toxic effects in organs and tissues. When overused, it can accumulate in the liver, skin, corneas, mucous membranes, kidneys, nails, gingiva, spleen, and other places. It can cause effects such as producing reactive oxygen species, and cell activation that is more toxic to tissue, which gradually leads to cell death [158].

## 5. Future Challenges

Synthetic methods involving fungus, bacteria, and other creatures are challenging since strain separation and growth are required. These processes are also difficult owing to the need to maintain the culture media, as well as the physical and chemical conditions. Plants are selected primarily because they are simple to extract and plentiful. As earlier explained, AgNP’s essential properties depend on their morphology and size. Therefore, future challenges lie in how these biological procedures can be used to produce other shapes such as triangular, cuboidal, truncated, ellipsoidal, pyramidal, decahedral, and oval shapes. Scaling up NP production from laboratory to commercial scale is not an easy task and has many difficulties and uncertainties. There are two further challenges. First, cost, dependability, waste, energy consumption, recycling potential, material safety, and hazard level should all be considered throughout manufacturing. Second, when nanomaterials scale up, their characteristics may alter. When working with huge volumes, the level of control may be compromised.

## 6. Summary

In this review paper, the research trends, worldwide use, synthesis, characteristics, and future challenges of Ag NPs have all been thoroughly assessed. Three methods are often employed to synthesize Ag-NPs: physical, chemical, and biological. The physical approach has several drawbacks, including high energy consumption, a large quantity of space needed, and a significant time to achieve thermal stability. Using a chemical technique, AgNPs may be easily prepared. On the other and, the usage of costly and dangerous chemicals is a major cause of environmental concern. Biological AgNP synthesis approaches are gaining popularity since they are ecologically friendly/green, cost-effective, and have no negative effects on the environment. Recent studies have revealed that AgNPs have good physical, chemical, biological, electrical, optical, thermal, and catalytic properties, making them appropriate for a wide range of essential applications. Concurrently, it IS crucial to acknowledge that AgNPs are dangerous, which must be considered when they are used in consumer items. Green synthesis should take into account three factors: simplicity, time consumption, and cost. It is also worth considering how this process may be enhanced to create shapes other than spheres. AgNPs discharged into the environment should be investigated from their origins, techniques, and transportation through their effects, utilizing better prototypes than those now available.

## Figures and Tables

**Table 1:** Synthesis of AgNPs from various bacterial species.

Year	S/N	Bacterial Species	Method	Size (nm)	Morphology	References
2021	1.	*Serratia nematodiphila*	Intracellular	10–31	Spherical	[18]
	2.	*Cyanobacteria Spirulina platensis and actinobacteria Streptomyces* spp. *211A*	Intracellular	7–15	-	[19]
	3.	*Klebsiella pneumonia*	Extracellular	1–6	Spherical	[20]
	4.	*Bacillus indicus*	Extracellular	-	Spherical	[21]
	5.	*Bacillus subtilis (MTCC441)*	Intracellular	10–100	Spherical	[22]
	6.	*Endosymbiotic Bacterium*	Intracellular	10–60	Cubic, spherical, hexagonal, crystalline, and oval	[23]
	7.	*Penicillium glabrum (MTCC 1985)*	Extracellular	26–32	Spherical	[24]
	8.	*Bacillus strain CS 11*	Extracellular	45 ± 0.15	FCC, Spherical	[25]
2020	9.	*Marine Ochrobactrum* sp.	Intracellular	35–85	Spherical	[26]
	10.	*Exiguobacterium mexicanum*	Extracellular	5–40	-	[27]
	11.	*Actinobacteria*	Intracellular	5–50	Spherical	[28]
	12.	*Lactobacillus strains*	Intracellular	15–500	Cluster triangular, hexagonal, and crystalline	[29]
	13.	*Pseudomonas proteolytica, Bacillus cecembensis*	Extracellular	6–13	Spherical	[30]
	14.	*Rhodococcus* sp.	Intracellular	5–50	Spherical	[26]
	15.	*Bacillus* sp.	Extracellular	5–15	Crystalline	[31]
	16.	*Bacillus licheniformis*	Extracellular	8–63	Spherical	[32]
	17.	*Shewanellao neidensis*	Intracellular	4 ± 1.5	Spherical	[33]
	18.	*Gluconacetobacter xylinus*	Intracellular	40–100	Spherical	[34]
	19.	*Bacillus subtilis*	Extracellular	5–60	Spherical	[35]
	20.	*Nocardiopsissp.MBRC-1*	Intracellular	45 ± 0.15	Spherical	[36]
	21.	*Pseudomonas stutzeri AG259*	Extracellular	35–200	Cluster equilateral triangular, and hexagonal	[37]
2019	22.	*Klebsiella pneumonia, Escherichia coli,* and *Enterobacter cloacae*	Extracellular	28–122	Spherical	[38]
	23.	*Aeromonas* sp. *THG-FG1.2*	Extracellular	8–16	fcc spherical	[39]
	24.	*Escherichia coli DH5a*	Extracellular	10–100	Spherical	[40]
	25.	*Pseudomonas putida NCIM 2650*	Extracellular	70	Spherical	[41]
	26.	*Vibrio alginolyticus*	Intracellular	50–100	Crystalline, spherical	[42]
	27.	*Lactobacillus casei*	Intracellular	20–50	Spherical	[43]
	28.	*Deinococcus radiodurans*	Extracellular	4–50	Spherical	[44]
	29.	*Bacillus pumilus, B. persicus,* and *B. licheniformis*	Extracellular	77–92	Spherical	[41]
	30.	*Staphylococcus aureus*	Extracellular	160–180	Spherical	[45]

**Table 2:** Synthesis of metallic NPs from various fungal species.

Year	S/N	Fungal Species	Method	Size	Morphology	References
2021	1.	*Endophytic fungus*	Intracellular	10–25	Polydispersedspherical, hexagonal, and spherical	[49]
	2.	*Trichoderma viride*	Extracellular	5–40	Spherical	[50]
	3.	*Schizophyllum commune*	Intracellular and extracellular	51–93	Spherical	[51]
	4.	*Humicola* sp.	Extracellular	5–25	Spherical	[52]
	5.	*Penicillium citrinum*	Extracellular	-	Uniform spherical	[53]
	6.	*Rhizopus stolonifer*	Extracellular	9.47	Spherical	[54]
	7.	*Cladosporium cladosporioides*	Intracellular	10–100	Spherical	[55]
	8.	*Fusarium semitectum*	Extracellular	1–50	Ellipsoid, polydispersed spherical	[56]
	9.	*Filamentous fungus*	Extracellular	58.35 ± 17.88	-	[57]
	10.	*Aspergillus flavus*	Extracellular	8.92	-	[58]
	11.	*Cladosporium sphaerospermum*	Extracellular	15.1 ± 1	Spherical	[55]
	12.	*Arthroderma fulvum*	Intracellular	20.56	Spherical	[59]
	13.	*Sclerotinia sclerotiorum MTCC 8785*	Extracellular	10–15	Spherical	[60]
2020	14.	*Penicillium brecompactum*	Intracellular	23–105	Crystalline spherical	[61]
	15.	*Rhizoctonia solani*	Intracellular	2–22	Spherical	[62]
	16.	*Rhizopus nigricans*	Extracellular	35–40	Round	[61]
	17.	*Alternaria alternate*	Extracellular	32.5	Polydispersed, spherical	[63]
	18.	*Aspergillus niger*	Extracellular	1–20	Polydispersed, spherical	[64]
	19.	*Penicillium hrysogenumad Aspergillus oryzae*	Extracellular	6–100, 14–76	Spherical	[65]
	20.	*Cryphonectria* sp.	Extracellular	30–70	-	[66]
	21.	*Penicillium* sp.	Extracellular	25–30	Spherical	[67]
	22.	*Penicillium* sp.	Extracellular	58.35 ± 17.88	-	[68]
	23.	*Aspergillus fumigates*	Extracellular	5–25	Spherical	[69]
2019	24.	*Guignardia mngifera*	Extracellular	5–30	Spherical	[70]
	25.	*Cariolus versicolor*	Intracellular	25–75	Spherical	[71]
	26.	*Duddingtonia flagrans*	Extracellular	30–409	Spherical	[72]
	27.	*Isaria fumosorosea*	Extracellular	51.31–111.02	Spherical	[73]
	28.	*Penicillium purpurogenum*	Intracellular	8–10	Spherical	[70]
	29.	*Fusarium solani*	Extracellular	5–35	Spherical	[15]
	30.	*Trichoderma harzianum*	Extracellular	34.77	Ellipsoidal, spherical	[74]
	31.	*Aspergillus fumigates*	Extracellular	5–25	Spherical	[72]
	32.	*Endophytic fungus*	Extracellular	25–30	Spherical	[75]
	33.	*Phoma glomerata*	Extracellular	60–80	Spherical	[15]
	34.	*Trichoderma reesei*	Extracellular	5–50	Random	[76]
	35.	*Fusarium acuminatum*	Extracellular	13	Spherical	[77]

**Table 3:** Ag NPs synthesized from diverse plants.

Year	S/N	Plant Name	Size (nm)	Morphology	References
2021	1.	*Phaseolus vulgaris*	10–20	Spherical	[79]
	2.	*Ficus Benjamina*	20–30	–	[80]
	3.	*Magnolia Kobus*	50–500	FCC	[20]
	4.	*Pinus thunbergii*	5–50	Triangular, hexagonal	[81]
	5.	*Ficus panda*	12–36	Spherical	[82]
	6.	*Dalbergia sissoo*	5–55	Spherical	[83]
	7.	*Musa balbisiana, Azadirachta indica, and Ocimum tenuiflorum*	100	Spherical, triangular, and cuboidal	[84]
	8.	*Buniumpersicum*	20–50	Spherical	[85]
	9.	*Acalypha Indica*	20–30	-	[86]
	10.	*Medicago Sativa*	2–20	Spherical	[87]
	11.	*Sesuvium portulacastrum* L.	5–20	Spherical	[88]
	12.	*Cyamopsis tetragonaloba*	8	Spherical	[89]
	13.	*Pine, persimmon, ginkgo, magnolia, and Platanus*	15–500	_	[90]
	14.	*Rosa Damascena*	-	Spherical	[91]
2020	15.	*Camellia Sinensis*	4	Spheroidal	[92]
	16.	*Sorghum bicolor*	10	Spheroidal	[93]
	17.	*Jatropha gossypifolia*	62	Spherical	[94]
	18.	*Coffee Arabica*	20–30	Spherical, Ellipsoidal	[41]
	19.	*Prunus yedoensis*	20–70	Spherical and oval	[95]
	20.	*Emblica Officinalis*	10–20	-	[96]
	21.	*Vitex negundo*	10–30	Spheroidal	[30]
	22.	*Cinnamomum camhora*	55–80	Triangular or spherical	[97]
	23.	*Mimosa pudica*	25–60	Spherical	[98]
	24.	*Camellia Sinensis*	20	Spheroidal	[92]
	25.	*Euphorbia lacteal*	186	Spherical	[99]
	26.	*Azadirachta Indica*	50–100	Spherical	[100]
	27.	*Morinda citrifolia*	30–55	Spherical	[101]
	28.	*Jatropha curcas*	10–20	Spherical	[97]
	29.	*Bauhinia variegate*	38–65	Spherical, triangle, truncated triangles, and decahedrons	[102]
	30.	*Pinus thunbergii*	20–100	Spheroidal	[103]
	31.	*Pulicaria glutinosa*	40–60	Spherical	[28]
	32.	*Nyctanthes arbor-tristis*	50–80	Spherical	[104]
	33.	*Terminalia chebula*	25	Spherical, ovoid	[105]
	34.	*Dioscorea bulbifera*	8–20	FCC	[106]
	35.	*Elaeagnus latifolia*	30–50	Spherical	[107]
	36.	*Vigna* sp. L.	24.35	Spherical	[108]
	37.	*Piper nigrum*	5–50	FCC	[109]
2019	38.	*Musa acuminata*	-	Agglomerated form	[110]
	39.	*Amaranthus retroflexus*	10–32	Spherical	[111]
	40.	*Tribulus Terrestris* L.	16–28	Spherical	[112]
	41.	*Cassia auriculata*	20–40	Spherical	[113]
	42.	*Adenium obesum*	10–30	Spherical	[40]
	43.	*Coleus aromaticus*	40–50	Spherical	[114]
	44.	*Artocarpus heterophyllus Lam*	10.78	Irregular	[115]
	45.	*Vigna radiate*	5–30	Spherical, oval	[116]
	46.	*Zingiber officinale*	10–20	-	[117]
	47.	*Lantana Camara*	14–27	Spherical	[118]
	48.	*Aloe vera*	20	Spherical	[119]
	49.	*Hevea brasiliensis*	2–100	Spherical	[120]
	50.	*Dodonaea viscosa*	16	Spheroidal	[121]
	51.	*Murraya koenigii*	20–35	Spheroidal	[15]
	52.	*Jatropha curcas*	73	Spherical	[122]
	53.	*Pedilanthus tithymaloides*	123	Spherical	[46]
	54.	*Euphorbia prostrate*	25–80	Rod	[123]
	55.	*Syzygium aromaticum*	-	-	[124]
	56.	*Tinospora cordifolia*	55–80	Aggregated	[125]
	57.	*Solanum torvum*	14	Spheroidal	[126]
	58.	*Murraya koenigii*	10–25	Spheroidal	[15]
	59.	*Ocimum tenuiflorum*	7–15	Spherical and ovoid	[75]

**Table 4:** Ag NPs prepared via bacteria, fungi, and plants.

Year	S/N	Organism	Biological Entity	Size (nm)	Morphology	Method	References
2021	1.	Fungi	*Penicillium* sp.	25	Spherical	Agar well diffusion method	[53]
	2.	Fungi	*Arthroderma fulvum*	15.5	Spherical	Brothmicro-dilution method	[18]
	3.	Fungi	*Penicillium aculeatum*	4–55	Spherical	Disk diffusion method	[37]
	4.	Bacteria	*Acinetobacter baumannii*	37–168	Spherical	Broth micro-dilution method	[136]
	5.	Plant	*Artocarpus altilis*	20–45	Spherical	Agar well diffusion method	[81]
	6.	Plant	*Convolvulus arvensis*	28	Spherical	Disc diffusion and broth macro-dilution method	[137]
	7.	Plant	*Erythrina suberosa*	15–34	Spherical	Agar cup and broth micro-dilution methods	[37]
	8.	Plant	*Psidium guajava*	20–25	Spherical	Agar well diffusion 1method	[89]
	9.	Plant	*Nelumbo Nucifera*	12.9	Quasi–Spherical	Broth dilution method	[138]
	10.	Plant	*Boerhaavia diffusa*	25	Spherical	Agar well diffusion	[20]
	11.	Plant	*Alpinia katsumadai*	12.6	Quasi-Spherical	Broth dilution method	[104]
	12.	Fungi	*Curvularia lunata*	64.3	Spherical	Disk diffusion assay	[139]
	13.	Fungi	*Pleurotus ostreatus*	10–40	Spherical	Disk diffusion and broth micro-dilution methods	[140]
2020	14.	Fungi	*Rhodotorula glutinis*	15–220	Spherical	Agar well diffusion and broth microdilution methods	[109]
	15.	Bacteria	*Pseudomonas deceptionensis*	127	Spherical	Agar well diffusion method	[141]
	16	Bacteria	*Acinetobacter baumannii*	37–168	Spherical	Broth micro-dilution method	[142]
	17.	Bacteria	*Phenerochaete* *Chrysosporium*	-	Spherical and oval	Agar well diffusion method	[143]
	18.	Bacteria	*Bacillus endophyticus*	4.8–6.6	Spherical	Agar well diffusion method	[144]
	19.	Plant	*tea*	10–20	Spherical	Disk and broth dilution methods	[145]
	20.	Plant	*Eucalyptus globules*	1.9–4.3	Spherical	Agar well diffusion and broth dilution methods	[109]
	21.	Plant	*Mimusops elengi*	55–83	Spherical	Disk diffusion method	[146]
2019	22.	Fungi	*Saccharomyces cerevisiae*	5–50	Spherical	Brothmicro-dilution method	[147]
	23.	Fungi	*Guignardia mangiferae*	5–30	Spherical	Agar well	[70]
	24.	Fungi	*Penicillium polonicum*	10–15	Spherical	Agar well diffusion	[148]
	25.	Fungi	*Raphanus sativus*	4–30	Spherical	Disk diffusion method	[149]
	26.	Fungi	*Ganoderma applanatum*	133	Spherical	Agar welldiffusionmethod	[150]
	27.	Bacteria	*Weissella oryzae*	150	Spherical	Disk diffusion method	[43]
	28.	Bacteria	*Ochrobactrum anthropi*	38–85	Spherical	Agar well diffusion method	[46]
	29.	Plant	*Elephantopus scaber*	37	Spherical	Agar well diffusion method	[46]
	30.	Plant	*Ocimum sanctum*	~15	Spherical	Disc and dilution approach	[151]
	31.	Plant	*Musa paradisiacal*	23.7	Spherical	Agar well diffusion and broth dilution approaches	[46]
	32.	Plant	*Dalbergia spinosa*	18	Spherical	Disk diffusion and broth microdilution methods	[15]
	33.	Plant	*Emblica officinalis*	15	Spherical	Disk diffusion method	[152]

## Data Availability

Not applicable.
